# Buckling Resistance of Two-Segment Stepped Steel Columns

**DOI:** 10.3390/ma14041046

**Published:** 2021-02-23

**Authors:** Bartłomiej Fliegner, Jakub Marcinowski, Volodymyr Sakharov

**Affiliations:** Institute of Civil Engineering, University of Zielona Góra, 65-516 Zielona Góra, Poland; fliegner.b@gmail.com (B.F.); j.marcinowski@ib.uz.zgora.pl (J.M.)

**Keywords:** stepped columns, critical force, buckling resistance, analytical formulae, experimental tests, numerical simulations

## Abstract

Columns of stepwise variable bending stiffness are encountered in the engineering practice quite often. Two different load cases can be distinguished: firstly, the axial force acting only at the end of the column; secondly, besides the force acting at the end, the additional force acting at the place where the section changes suddenly. Expressions for critical forces for these two cases of loading are required to correctly design such columns. Analytical formulae defining critical forces for pin-ended columns are derived and presented in the paper. Derivations were based on the Euler-Bernoulli theory of beams. The energetic criterion of Timoshenko was adopted as the buckling criterion. Both formulae were derived in the form of Rayleigh quotients using the Mathematica^®^ system. The correctness of formulae was verified based on one the of transcendental equations derived from differential equations of stability and presented by Volmir. Comparisons to results obtained by other authors were presented, as well. The derived formulae on the critical forces can be directly used by designers in procedures leading to the column’s buckling resistance assessment. The relatively simple procedure leading to buckling resistance assessment of steel stepped columns and based on general Ayrton-Perry approach was proposed in this work. The series of experimental tests made on steel, stepped columns and numerical simulations have confirmed the correctness of the presented approach.

## 1. Introduction

**Figure 1:**
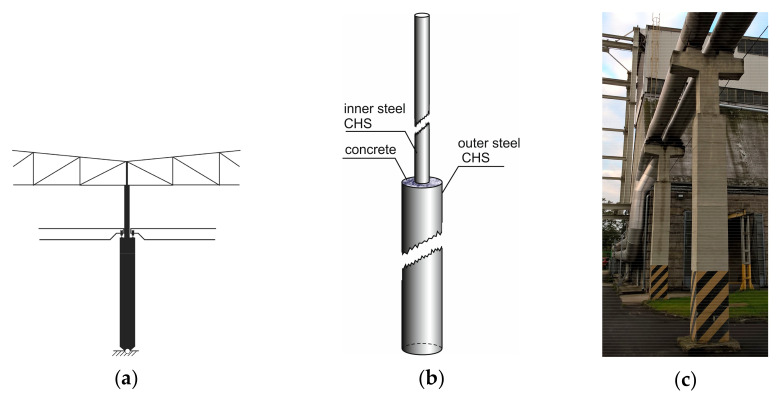
Examples of two-segment stepped columns: (**a**) An inner column in industrial multi-nave steel mill building; (**b**) CHS column filled by concrete in lower part; (**c**) Two stepped, industrial column.

Columns of stepwise variable bending stiffness are quite often encountered in the engineering practice. Steel, concrete or hollow columns filled by the concrete to a specific level (cf. Figure 1) are examples of columns of stepwise variable sections.

Columns of steel mill buildings are the typical example of stepped columns. Such a column with a crane girder plays double role: supporting the roof structure and carrying loadings from the crane. The exemplary solution of inner columns in industrial multi-nave steel mill buildings is shown in Figure 1a. In this case when the crane girder is present, the column is loaded at the end and additionally at the place of the sudden change of section. In Figure 1b the typical solution of the two step column, which is composed of two coaxial steel tubes filled with a concrete, is shown. Figure 1c presents an example of stepped reinforced concrete column.

**Figure 2:**
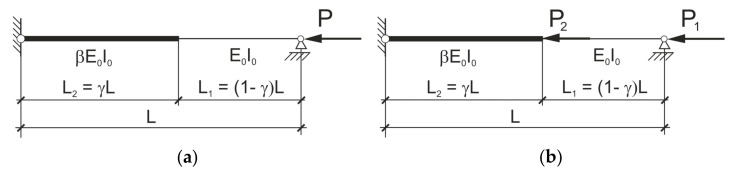
Columns of stepwise variable bending stiffness loaded: (**a**) By force *P* applied at the end; (**b**) By forces *P*_1_ and *P*_2_.

The correct design of stepped columns requires knowledge of the critical force causing the bending buckling. In the widely available literature referring to the mechanics or structural problems there is a shortage of closed formulae describing the critical force of compressed rods of the stepwise variable bending stiffness like these shown in Figure 2.

The first analytical solutions of the buckling problem of stepped columns were published in [1,2]. The critical force was not obtained explicitly but as a result of the solution of the transcendental equation that had to be solved for each specific case of the strut. Other attempts of determination of the critical force or equivalent length of stepped columns were presented in [3,4,5,6]. The problem has attracted attention of researchers also later (cf. [7,8]) and contemporary (cf. [9,10,11]). Toosi et al. [12] for calculating the buckling load of stepped columns proposed to use a method based on modified buckling mode shape of tapered structure and perturbation theory. In a case of multi-bay frame Tian et al. [13] proposed the method, which can be applied to frames composed of stepped (or prismatic) columns. Asquez and Riddel [9] present an effective numerical model based on Przemieniecki [14] approach. Pinarbasi et al. [10] determine the critical force of stepped columns using the variational iteration method (VIM). Simao et al. [15,16], the buckling problem of stepped column have reduced to a discrete, two degrees of freedom system. The eigenvalue problem of matrix 8 × 8 gives the searched critical value of the load parameter. The results for various boundary conditions and various parameters describing the problem under consideration are presented in these papers in the form of many tables.

The main disadvantage of solutions presented in the above mentioned works is the lack of explicit formulae that would be the most convenient for engineers designing stepped columns. The present work is free of this drawback. Authors present the solution of stepped column’s buckling problems, which is expressed by the explicit formula in which all the parameters describing the stepped column made of any material are included. The first proposal of this solution was published in [17].

The correctness of derived formulae was verified experimentally on two-segment, steel stepped columns. Numerical simulations, carried out by means of Abaqus and COSMOS/M systems (cf. [18,19,20,21]), have confirmed the correctness of derived formulae, as well.

Buckling forces and corresponding equivalent lengths of both segments of stepped columns are required to the assessment of buckling resistance of the column. This problem was considered first by Barnes and Mangelsdorf [22] and then by Castiglioni [23,24]. Some aspects of the buckling resistance of stepped columns were discussed in [25,26,27]. The most comprehensive studies referring to the buckling resistance of stepped column were presented in works [28,29].

In the present work the comparatively easy approach, leading to the buckling resistance assessment of stepped steel columns, was proposed. The proposed procedure was based on the original Ayrton-Perry approach (cf. [30]) in which the assumed amplitude of the bow imperfection was used. Experimental tests performed on two-segment stepped, steel columns have positively verified correctness of the proposed approach. Numerical simulations in which material parameters obtained in performed material tests were used, have confirmed the correctness of the proposed procedure as well.

## 2. Determination of Critical Forces

### 2.1. The Column Loaded by a Force Applied at the End

Let us consider first the pin-ended column of stepwise variable bending stiffness shown in Figure 2a. The critical force of the column shown in Figure 2a one can determine solving the transcendental equation (cf. Volmir [2]). This task is not easy for a practicing engineer yet. The closed formula for this case and for arbitrary values of coefficients *β* and *γ* is required and such a formula was derived in this work.

**Figure 3:**
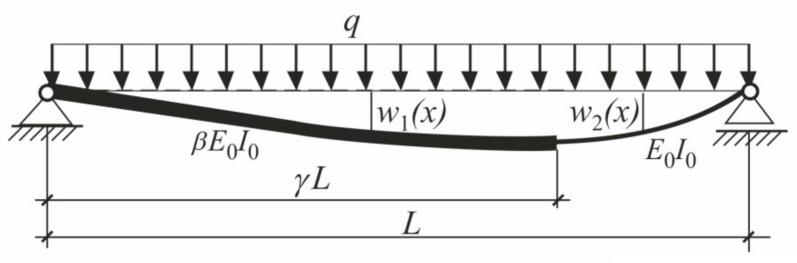
Deflections *w*_1_(*x*) and *w*_2_(*x*) caused by the uniformly distributed load.

To derive the closed analytical expression for the critical force the energetic criterion of Timoshenko [1] was applied. The deflection shape caused by the lateral uniform loading was utilized as the mode of the bending buckling. Due to different bending stiffnesses the deflection shape is described by the two different functions within the first and the second intervals (comp. Figure 3). The functions shown in Figure 3 were derived in general analytical form in which parameters *β* and *γ* were utilized.

The energetic criterion of Timoshenko which expresses the identity between the increase of elastic energy of bending and the increment of work done by external forces leads to the following relation:

(1) $$\frac{1}{2}\int_{0}^{\gamma L}\frac{P^{2}w_{1}^{2}}{\beta E_{0}I_{0}}dx+\frac{1}{2}\int_{\gamma L}^{L}\frac{P^{2}w_{2}^{2}}{E_{0}I_{0}}dx=\frac{1}{2}\int_{0}^{\gamma L}{Pw^{\prime }}_{1}^{2}dx+\frac{1}{2}\int_{\gamma L}^{L}{Pw^{\prime }}_{2}^{2}dx,$$

from which the subsequent formula on searched *P_kr_* can be obtained:

(2) $$P_{kr}=E_{0}I_{0}\frac{\int_{0}^{\gamma L}{w^{\prime }}_{1}^{2}dx+\int_{\gamma L}^{L}{w^{\prime }}_{2}^{2}dx}{\frac{1}{\beta }\int_{0}^{\gamma L}w_{1}^{2}dx+\int_{\gamma L}^{L}w_{2}^{2}dx},$$

where *E*_0_*I*_0_ is the bending stiffness of the column within the second segment. The notation (*w_i_*)’ means the first derivative of the function *w_i_* with respect to *x*.

The final formula was obtained in the form of the Rayleigh quotient and all the derivations were carried out by means of the Mathematica^®^ system (cf. [31]), which allows performing symbolic derivations. Standard commands available in this system were used to this end.

Assuming that both parameters *β* and *γ*, the total length of the rod *L* and the bending stiffness *E*_0_*I*_0_ are known, the critical force can be expressed by the following formula:

(3) $$P_{kr}=\frac{F_{g}}{F_{d}}\frac{E_{0}I_{0}}{L^{2}}$$

where:

(4) $$\begin{array}{c} F_{g}\left(\beta ,\gamma \right)=18\beta [\beta^{2}\left(315\gamma^{3}+255\gamma^{2}+85\gamma +17\right){\left(\gamma -1\right)}^{5}-70\beta \gamma^{3}\left(9\gamma^{2}-9\gamma -4\right)\\ \left(\gamma -1{)}^{3}+\gamma^{5}\left(315\gamma^{3}+1540\gamma -1200\gamma^{2}-672\right)\right] \end{array}$$

(5) $$\begin{array}{c} F_{d}(\beta ,\gamma )=\;\beta^{3}\;(1890\;\gamma^{4}\;+\;1890\;\gamma^{3}\;+868\;\gamma^{2}\;+\;217\;\gamma \;+\;31)\;{\left(\gamma -1\right)}^{7}\;+\\ \;42\;\beta \;\gamma^{5}\;(135\;\gamma^{3}\;+\;149\;\gamma \;-\;315\;\gamma^{2}\;+\;48)\;{\left(\gamma -1\right)}^{3}\;-\;\\ 42\;\beta^{2}\;{\left(\gamma \;-\;1\right)}^{5}\;\gamma^{3}\;(135\;\gamma^{3}\;-\;76\;\gamma \;-\;90\;\gamma^{2}\;-\;17)\;+\;\\ \gamma^{7}\;(9450\;\gamma^{3}\;+\;15183\;\gamma \;-\;17878\;\gamma^{2}\;-\;1890\;\gamma^{4}\;-4896) \end{array}$$

**Figure 4:**
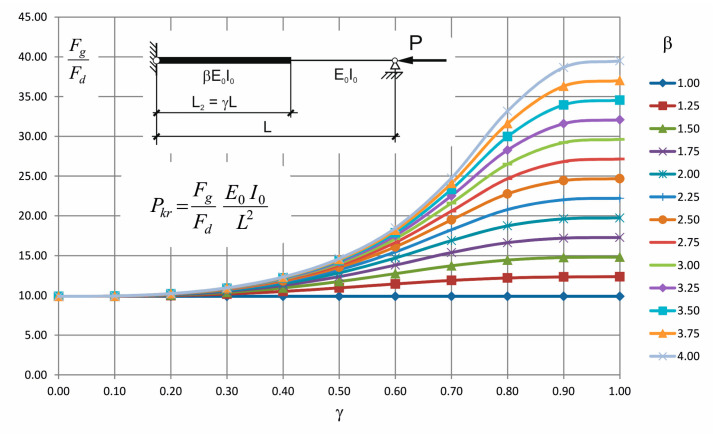
The ratio *F_g_/F_d_* for different values of *β* and *γ*.

The ratio *F_g_/F_d_* was presented in Table 1 for typical range of parameters *β* and *γ*. Additionally, the family of graphs illustrating this relationship is presented in Figure 4.

To verify the correctness of the derived formula the analytical solution presented by Volmir [2] is used. The transcendental equation for this particular case can be reduced to the following:

(6) $$\frac{1}{tg[x\left(1-\gamma \right)]}\;=\;\frac{-1}{\sqrt{\beta }tg\left[x\frac{\gamma }{\sqrt{\beta }}\right]}$$

in which:

(7) $$x\;=\;\sqrt{\frac{P_{}}{E_{0}I_{0}}}L\;$$

To check the correctness of the derived formula the following case is considered: *β* = 3.75 and *γ* = 0.2. The solution of Equation (6) gives *x* = 3.1950, and from Equation (7) one obtains $P_{kr}\;=\;10,208\frac{E_{0}I_{0}}{L^{2}}\;$. Equation (3) gives multiplier 10.21 (look also at Table 1); it means that correspondence of the derived formula with the exact solution is excellent.

Other examples of the practical application of Equation (1) were presented in [14] where the numerical approach was adopted, as well.

**Figure 5:**
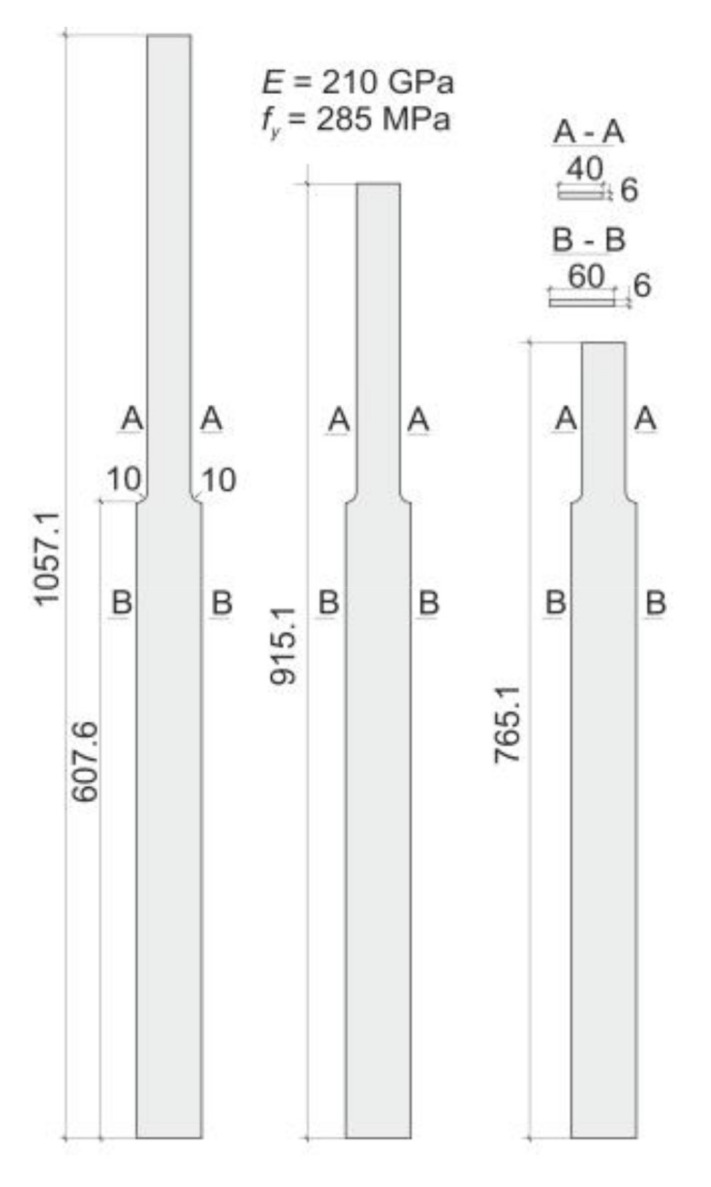
Columns modeled numerically.

Some particular cases of columns, shown in Figure 2a, were modeled numerically. Material and geometrical parameters of considered columns are presented in Figure 5.

According to adopted data: $E_{0}I_{0}=1.512\cdot 10^{8}$ Nmm^2^, *β* = 1.5, Abaqus and COSMOS/M systems were used and domains of columns were modeled using the shell elements of S4 and SHELL4 families in Abaqus and COSMOS/M systems respectively (cf. [18,19,20,21]). Critical forces, obtained as results of linear buckling analyses (LBA), were presented in Table 2 and compared to results obtained by analytical approach. Values shown in the column 4 are results of solution of transcendental Equation (6). These are exact solutions of considered buckling problems.

Critical forces, obtained experimentally and presented in column 7 of Table 2, will be discussed in Section 4 of this paper.

Results presented in Table 2 confirm correctness of the derived formulae in reference to the considered particular cases of stepped columns.

### 2.2. The Column with Additional Force Acting at the Column’s Span

The considered case is presented in Figure 2b. Let forces *P_1_* and *P_2_* be related by the relation: *P*_2_= *αP*_1_, where *α* is given. The analogous procedure like this, described in the clause 2.1, leads to the following formula on the critical force:

(8) $$P_{1,kr}\;=\;\frac{P_{g}}{P_{d}}\cdot \frac{E_{0}I_{0}}{L^{2}}$$

in which:

(9) $$\begin{array}{l} P_{g}\;=\;18\;\beta \;\{\gamma^{5}\;[672-1540\;\gamma \;+\;1200\;\gamma^{2}\;-\;315\;\gamma^{3}\;+\;\alpha \;(672\;-2100\;\gamma \;+\;2600\;\gamma^{2}\;-\;\\ 1470\;\gamma^{3}\;+\;315\;\gamma^{4})]\;-\;70\;\beta \;[\alpha (\gamma -1)-1]\;{\left(\gamma \;-\;1\right)}^{3}\;\gamma^{3}\;(9\;\gamma^{2}\;-9\;\gamma \;-4)\;+\;\\ \beta^{2}\;{\left(\gamma -1\right)}^{5}\;[315\;\alpha \;\gamma^{4}\;-\;105\;(\alpha \;+3)\;\gamma^{3}\;-5\;(35\;\alpha \;+51)\;\gamma^{2}\;-\;5\;(7\;\alpha \;+\;17)\;\gamma \;-\;17]\}, \end{array}$$

(10) $$\begin{array}{l} P_{d}\;=\;-\beta^{3}\;{\left(\gamma \;-\;1\right)}^{7}\;[31\;+\;7\;(31\;+\;18\;\alpha )\;\gamma \;+\;14\;\gamma^{2}\;(62\;+\;63\;\alpha \;+15\;\alpha^{2})\;+\\ 42\;\gamma^{3}\;(45\;+\;51\;\alpha \;+\;20\;\alpha^{2})\;-\;210\;\gamma^{4}\;(2\alpha^{2}\;-\;3\;\alpha \;-\;9)\;-\;1260\;\alpha \;\gamma^{5}(2\;\alpha \;+\;3)\;+\;\\ 1890\;\alpha^{2\;}\;\gamma^{6}]\;+\;42\;\beta^{2}\;\gamma^{3}\;{\left(\gamma \;-\;1\right)}^{5}\;[135\;\gamma^{3}\;-90\;\gamma^{2}\;-\;76\;\gamma -\;17+\;\\ 5\;\alpha^{2}\;{\left(\gamma \;-\;1\right)}^{2}\;(27\;\gamma^{3}\;-\;21\;\gamma^{2}\;-\;13\;\gamma \;-\;1)\;-\;\alpha \;(270\;\gamma^{4}\;-\;465\;\gamma^{3}\;+\;54\;\gamma^{2}\;+\\ \;119\;\gamma \;+\;22)]\;-42\;\beta \;\gamma^{5}\;{\left(\gamma \;-\;1\right)}^{3}\;[135\;\gamma^{3}\;-315\;\gamma^{2}\;+\;149\;\gamma \;+\;48\;-\\ 3\;\alpha \;(90\;\gamma^{4}\;-\;295\;\gamma^{3}\;+303\;\gamma^{2}\;-\;70\;\gamma \;-\;32)+\;\alpha^{2}\;(135\;\gamma^{5}\;-\;570\;\gamma^{4}\;+\;880\;\gamma^{3}\;-\;\\ 554\;\gamma^{2}\;+\;61\;\gamma \;+\;48)]\;+\;\;\gamma^{7}\;[1890\;\gamma^{4}\;-\;9450\;\gamma^{3}\;+17878\;\gamma^{2}\;-\;15183\;\gamma \;+\;4896\;-\;\\ 2\;\alpha \;(1890\;\gamma^{5}\;-\;11025\;\gamma^{4}\;+\;26019\;\gamma^{3}\;-31234\;\gamma^{2}\;+\;19215\;\gamma \;-\;4896)\;+\;\\ \alpha^{2}\;(1890\;\gamma^{6}\;-\;12600\;\gamma^{5}\;+\;35490\;\gamma^{4}\;-\;54348\;\gamma^{3}\;+\;47950\;\gamma^{2}\;-\;23247\;\gamma \;+\;4896)]. \end{array}$$

These formulae can be entered into a table or illustrated in a form of nomogram plots from which, after a possible interpolation, the critical value of the force can be found.

Values of the ratio *P_g_/P_d_* for different values of *β* and *γ* and for *α* = 0.5, 1.0 and 2.0 are presented in Table 3, Table 4 and Table 5, respectively.

**Figure 6:**
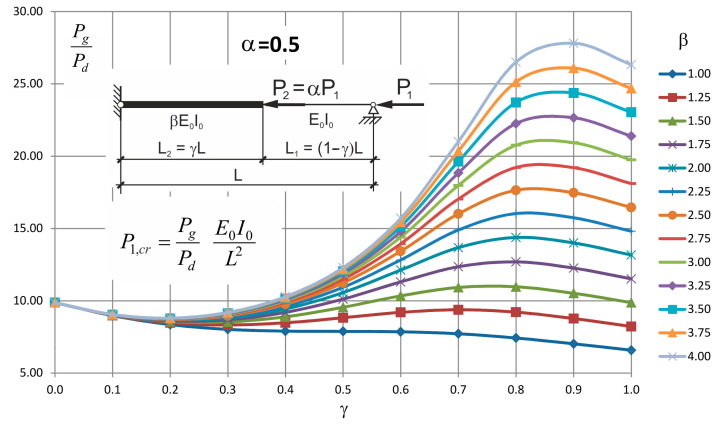
Values *P_g_/P_d_* for *α* = 0.5 and for different values of *β* and *γ*.

**Figure 7:**
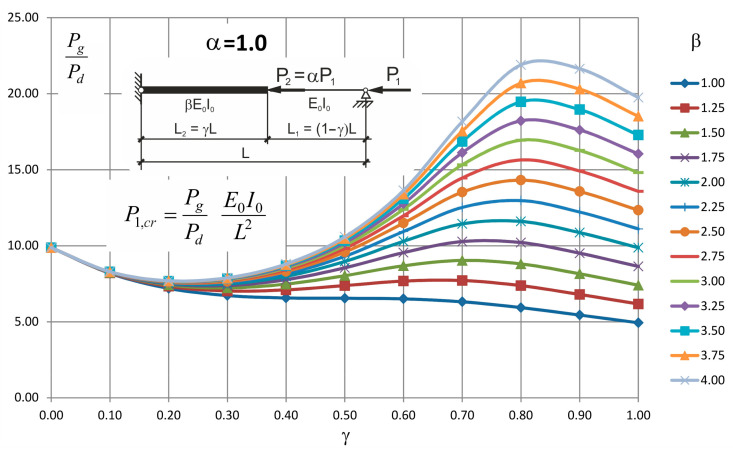
Values *P_g_/P_d_* for *α* = 1.0 and for different values of *β* and *γ*.

**Figure 8:**
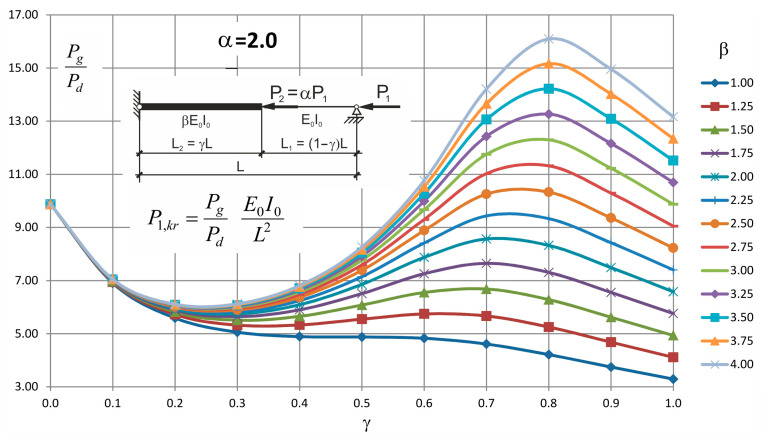
Values *P_g_/P_d_* for *α* = 2.0 and for different values of *β* and *γ*.

Plots obtained based on these values are presented in Figure 6, Figure 7 and Figure 8.

Looking at these graphs one can observe that always exists such a value of *γ* for which the critical force attains the maximum value and it is not the value of 1.0. It means that there is no need to strengthen the whole two step column to obtain its maximum buckling resistance.

To verify the correctness of the derived formula, the exact analytical solution presented by Volmir [2] is used. The transcendental equation, from which the exact value of the critical force can be determined, adopts in this case the following form (comp. [2]):

(11) $$\frac{k_{4}^{2}}{k_{1}^{2}}\;-\;\frac{k_{1}^{2}L\;+\;k_{4}^{2}L_{1}}{k_{1}\;tgk_{1}L_{1}}\;=\frac{k_{3}^{2}}{k_{2}^{2}}\;+\;\frac{k_{2}^{2}L\;-\;k_{3}^{2}L_{2}}{k_{2}\;tgk_{2}L_{2}}$$

where:

(12) $$k_{1}^{2}\;=\;\frac{P_{1}}{EJ_{1}},\;k_{2}^{2}\;=\;\frac{P_{1}\;+\;P_{2}}{EJ_{2}}\;,\;k_{3}^{2}\;=\;\frac{P_{2}}{EJ_{2}},\;k_{4}^{2}\;=\;\frac{\;P_{2}}{EJ_{1}}\;$$

The case defined in Figure 2b one can adjust to this equation introducing the following notation:

(13) $$EJ_{1}\;=\;E_{0}I_{0}\;,\;EJ_{2}\;=\;\beta E_{0}I_{0},\;L_{2}\;=\;\gamma L,\;L_{1}\;=\;\left(1-\gamma \right)L,\;P_{2}\;=\;\alpha P_{1}$$

In this case:

(14) $$k_{1}^{2}\;=\;\frac{P_{1}}{E_{0}I_{0}},\;k_{2}^{2}\;=\;\frac{P_{1}\;+\;\alpha P_{1}}{\beta E_{0}I_{0}}\;=\;\frac{\left(1+\alpha \right)}{\beta }k_{1}^{2},\;\;k_{3}^{2}\;=\;\frac{\alpha P_{1}}{\beta E_{0}I_{0}}\;=\;\frac{\alpha }{\beta }\;k_{1}^{2},\;k_{4}^{2}\;=\;\frac{\;\alpha P_{1}}{E_{0}I_{0}}\;=\;\alpha \;k_{1}^{2}$$

The new unknown *x* is defined in the following way:

(15) $$x\;=\;\sqrt{\frac{P_{1}}{E_{0}I_{0}}}L\;$$

and now:

(16) $$k_{1}^{2}\;=\;\frac{x^{2}}{L^{2}},\;k_{2}^{2}\;=\;\frac{\left(1+\alpha \right)}{\beta }\frac{x^{2}}{L^{2}},\;k_{3}^{2}\;=\;\frac{\alpha }{\beta }\;\frac{x^{2}}{L^{2}},\;k_{4}^{2}\;=\;\alpha \;\frac{x^{2}}{L^{2}}$$

The Equation (7) on the unknown x adopts the following form:

(17) $$\alpha \;-\;x\frac{1\;+\;\alpha \left(1-\gamma \right)}{tg[x\left(1-\gamma \right)]}\;=\frac{\alpha }{1+\alpha }\;+\;\frac{x}{\beta }\frac{1+\alpha \left(1\;-\;\gamma \right)}{\sqrt{\frac{1+\alpha }{\beta }}tg\left[x\gamma \sqrt{\frac{1+\alpha }{\beta }}\right]}$$

Solving this transcendental equation for given values of *α*, *β* and *γ* one can find the critical value of the force *P*_1_ from the relationship:

(18) $$P_{1}\;=\;x^{2}\frac{E_{0}I_{0}}{L^{2}}$$

It should be remembered that this force is always accompanied by the force *P_2_ = αP_1_*.

As an example of utilization of the transcendental Equation (17), the following case is considered: *α* = 0.5, *β* = 1.5, *γ* = 0.5. From Equation (17) one can obtain *x* = 3.08902. Hence, $P_{1}\;=\;9,5420\;\frac{E_{0}I_{0}}{L^{2}},\;P_{2}\;=\;4,7710\;\frac{E_{0}I_{0}}{L^{2}}$ and this is the exact solution.

For this particular case the derived Formula (8) gives the multiplier 9.55 (comp. Table 2). The error on the level of 0.1% confirms the high accuracy of the derived formula.

The obtained results were compared also with solutions presented by Pinarbasi et al. [10]. Formulae derived in the paper give results which compare favorably with results presented in [10] in which critical forces were presented as a result of numerical analysis only for discrete values of parameters defining the given problem. Advantage of the proposed analytical formulae is obvious: it allows calculation of critical forces for any values of parameters *α*, *β* and *γ* for the considered case of boundary condition.

The critical forces of columns considered in this clause were determined also numerically for some particular cases. The COSMOS/M and Abaqus systems were used to this end again. The same material and geometrical parameters as those shown in Figure 5 were adopted. The Critical forces for the case *α* = 2 (the force *P*_2_ = 2*P*_1_ applied at a distance 607.6 mm) are presented in Table 6 in which also results obtained in analytical approach (Equation (8)) are shown. In the column no. 4 the values resulting from the solution of transcendental Equation (18) are presented.

Results presented in Table 6 confirm correctness of the derived formulae in reference to the considered particular cases of stepped columns. The maximum error, in reference to the exact solution, does not exceeds 1% (it is equal 0.6%).

## 3. The compressive Resistance of Stepped Steel Columns

**Figure 9:**
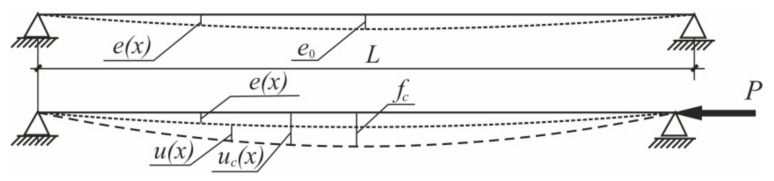
Deflections of the initially curved rod.

The resistance of the considered stepped steel columns can be assessed based on the classical Ayrton-Perry’s approach (cf. [30]). The initial bow imperfection with amplitude $e_{0}$ (Figure 9) in a form of one half-wave sine function is defined, as follows:

(19) $$e\left(x\right)=e_{0}\sin\frac{\pi x}{L}$$

The total deflection $u_{c}\left(x\right)$ can be obtained from the formula (cf. [1]):

(20) $$u_{c}\left(x\right)=f_{c}\sin\frac{\pi x}{L},\;f_{c}=\frac{e_{0}}{1-\frac{P}{P_{cr}}}$$

where $f_{c}$—total eccentricity in the middle section of the rod, *P_cr_*—critical buckling force.

The maximum longitudinal stresses at arbitrary cross section, defined by *x*, can be calculated from the formula:

(21) $$\sigma_{max}=\frac{P}{A\left(x\right)}+\frac{Pf_{c}}{W\left(x\right)}\sin\frac{\pi x}{L}\leq f_{y},$$

in which $A\left(x\right)$ and $W\left(x\right)$ are the cross-sectional area and the elastic section modulus, respectively. The quantity $f_{y}$ used in inequality (21) is the nominal yield strength for the giving steel grade according to EN1993-1-1.

Using (20) and taking the equality in (21) are obtains:

(22) $$\frac{P}{A\left(x\right)}+\frac{P}{W\left(x\right)}e_{0}\frac{P_{cr}}{P_{cr}-P}\sin\frac{\pi x}{L}=f_{y}$$

To convert Equation (22) to the form known from EN1993-1-1 (2005) the following notations are used:

(23) $$P\left(x\right)=\chi \left(x\right)\cdot A\left(x\right)\cdot f_{y},\;\hat{\lambda }\left(x\right)=\sqrt{\frac{f_{y}A\left(x\right)}{P_{cr}}}$$

where: *χ*(*x*) is the buckling reduction factor, $\hat{\lambda }\left(x\right)$ is the dimensionless measure of the slenderness, both dependent on *x* in this particular case.

It is worth mentioning that in contrary to provisions of EN1993-1-1 (2005) the buckling reduction factor *χ* and dimensionless slenderness *λ* are dependent on the current coordinate *x* in the presented approach.

Substituting (23) to Equation (22) and introducing the function Φ(*x*) defined as follows:

(24) $$\Phi \left(x\right)=\frac{1}{2}\left[1+\frac{A\left(x\right)}{W\left(x\right)}e_{0}\sin\frac{\pi x}{L}+{\hat{\lambda }}^{2}\left(x\right)\right]$$

one obtains the following:

(25) $$\chi^{2}\left(x\right)\cdot {\hat{\lambda }}^{2}\left(x\right)-\chi \left(x\right)\cdot 2\Phi \left(x\right)+1=0,$$

from which the searched reduction factor χ(*x*) is obtained in the form:

(26) $$\chi \left(x\right)=\frac{\Phi \left(x\right)-\sqrt{\Phi^{2}\left(x\right)-{\hat{\lambda }}^{2}\left(x\right)}}{{\hat{\lambda }}^{2}\left(x\right)}=\frac{1}{\Phi \left(x\right)+\sqrt{\Phi \left(x\right)-{\hat{\lambda }}^{2}\left(x\right)}}$$

which is consistent with formula (6.49) from EN 1993-1-1 (2005) valid for columns with the constant cross-section.

The column’s resistance $P_{ult}$ is determined by the smallest value of the expression:

(27) $$P_{ult}\left(x\right)=\chi \left(x\right)\cdot A\left(x\right)\cdot f_{y}$$

The value of ultimate force defined by formula (27) could be too high in some circumstances. For the safe design procedures, the additional partial coefficient $\gamma_{M}=1.1$ is proposed and the final formula for the design value of column’s buckling resistance is as follows:

(28) $$P_{Rd}\left(x\right)=\frac{1}{\gamma_{M}}\cdot \chi \left(x\right)\cdot A\left(x\right)\cdot f_{y}$$

The whole procedure can be easily inserted in a spreadsheet for every *x* from the interval 0 < *x* < *L* and in this way the smallest value of $P_{Rd}$ can be found.

The initial bow amplitude $e_{0}$ required in this procedure can be adopted according to the code recommendations. Following provisions inserted in EN 10219-2, EN 10210-2 (2006) and EN 1090-2 (2018), the $e_{0}$ can be adopted as *L*/750, and this value, guaranteeing the conservative assessment of columns resistance, was adopted in examples presented in the next section.

**Figure 10:**
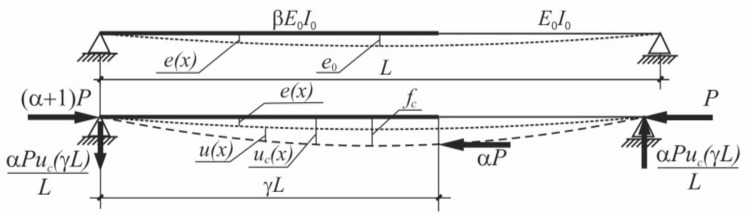
Deflections of the initially curved rod. The case with additional force in the column’s span.

The resulting formulae will be different for the case when the additional force is present in the column’s span (the case shown in Figure 2b and Figure 10). Assuming as before that the initial bow imperfection is present (comp. (19)), the expressions for bending moments within two segments of the bar shown in Figure 10 are as follows:

(29) $$M\left(x\right)=P\left(\alpha +1\right)u_{c}\left(x\right)-P\alpha \frac{u_{c}\left(\gamma L\right)}{L}x=Pf_{c}\sin\frac{\pi x}{L}\left[1+\alpha \left(1-\frac{x}{L}\frac{\sin\left(\gamma \pi \right)}{\sin\frac{\pi x}{L}}\right)\right]\;\;\mathrm{for}\;0<x\leq \gamma L,$$

(30) $$M\left(x\right)=Pu_{c}\left(x\right)+P\alpha \frac{u_{c}\left(\gamma L\right)}{L}x=Pf_{c}\sin\frac{\pi x}{L}\left[1+\alpha \left(1-\frac{x}{L}\right)\frac{\sin\left(\gamma \pi \right)}{\sin\frac{\pi x}{L}}\right]\;\;\mathrm{for}\;\gamma L\leq x<L.$$

Instead of Equation (21) the following condition can be written now:

(31) $$\sigma_{max}=\frac{P}{A\left(x\right)}+{\frac{M\left(x\right)}{W\left(x\right)}}_{y}\leq f_{y}$$

Assuming that both expressions (20) hold true and making the analogous derivations as before, the following expressions for functions Φ(*x*) are obtained:

(32) $$\Phi \left(x\right)=\frac{1}{2}\left\{1+\frac{A\left(x\right)}{W\left(x\right)}e_{0}\left[1+\alpha -\frac{\alpha }{L}\frac{\sin\left(\gamma \pi \right)}{\sin\frac{\pi x}{L}}x\right]\sin\frac{\pi x}{L}+{\hat{\lambda }}^{2}\left(x\right)\right\}\;\mathrm{for}\;0<x\leq \gamma L,$$

(33) $$\Phi \left(x\right)=\frac{1}{2}\left\{1+\frac{A\left(x\right)}{W\left(x\right)}e_{0}\left[1+\frac{\alpha }{L}\frac{\sin\left(\gamma \pi \right)}{\sin\frac{\pi x}{L}}x\right]\sin\frac{\pi x}{L}+{\hat{\lambda }}^{2}\left(x\right)\right\}\;\mathrm{for}\;\gamma L\leq x<L$$

The general forms of Equations (28)–(31) remain unchanged. As before, the ultimate force should be calculated at every column’s section *x* and the smallest value is the measure of the column’s compressive resistance. All the calculations can be carried out by means of the spreadsheet.

The compression resistances of columns considered in Section 2 will be determined now using derived formulae. Resistances of columns loaded at ends and loaded additionally at the section of the sudden cross-section change, calculated from Formulas (30) and (31), are presented in Table 7 and Table 8 and labeled as $P_{Rd}^{prop}$ Results of geometrically and materially nonlinear analyses, carried out by means of the Abaqus system, are presented in these tables as well. $P_{Rd}^{num}$ are the maximum values of force on the load-displacement paths obtained for the initial bow imperfection of amplitude *L*/750.

These results were obtained for the yield stress *f_y_* = 285 MPa, the value determined in material tests made on coupons cut from the same steel sheet from which the analyzed columns were made.

## 4. Experimental Tests and Numerical Simulations

**Figure 11:**
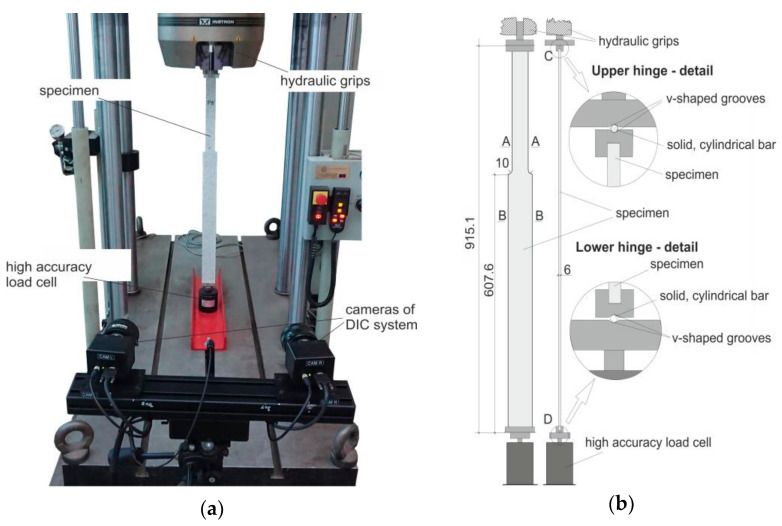
The test rig: (**a**) General view; (**b**) Details.

To confirm the correctness of the proposed method, experimental tests were carried out on steel specimens shown in Figure 5. Specimens were prepared by laser cut from the steel sheet of thickness 6 mm. Figure 11 shows the test rig with its most important details. The main part of the test rig is the frame of Instron testing machine. To accomplish the pin ended boundary conditions the special accessories were designed. Details of these additional elements, used in every test, are shown in Figure 11b.

The compressive force was generated by the downward movement of the upper hydraulic grips. The value of the force was measured by the load cell placed beneath the lower hinge of the specimen. Horizontal and vertical displacements of specimens during the test were measured by means of the noncontact optical DIC (digital image correlation) system. To this end, the specimen’s surface was covered by black, speckle pattern visible in Figure 11a.

It is worth mentioning that specimens did not exhibit any initial geometrical imperfections. The existing residual stresses (due to hot rolling of the steel sheet) were present inside specimens because they were not heat treated (annealed) before experiments.

**Figure 12:**
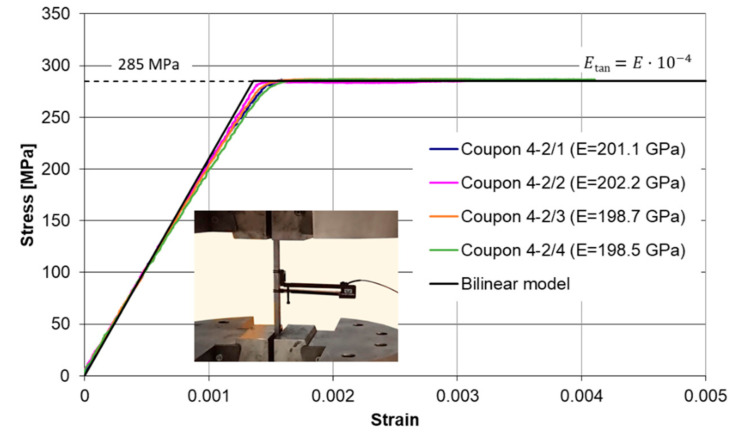
Material investigations of coupons cut from the Test Beam 4.

Material investigations on coupons cut from steel sheets were carried out and acquired material parameters were used in numerical simulations. (Exemplary stress-strain curves are shown in Figure 12).

Experimental tests proceeded as follows. The quasi-static (0.5 mm/min), downward movement of the upper grip was initiated. The accompanying forces and horizontal and vertical displacements of the specimen’s central zone were recorded at a rate of 5 samples per second.

Three specimens of each kind of columns shown in Figure 5 were examined. In some cases tests were repeated on the same specimen two or even three times provided they were limited to the elastic range.

**Figure 13:**
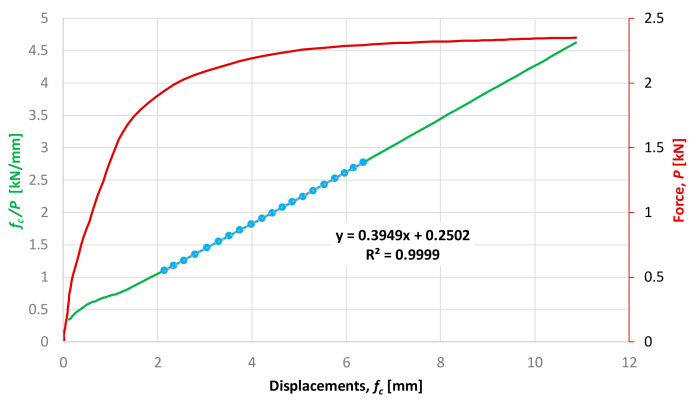
Specimen *L =* 915 mm. The initial part of the load deflection path and the Southwell’s plot.

The critical forces were determined experimentally by means of the Southwell method (comp. [1] Chapter 4) after choosing the initial portion of the load-displacement curve. An example of determination of critical force is presented in Figure 13.

According to the Southwell method, the critical force is the inverse of the slope coefficient of the *f*_c_/*P* versus *f_c_* characteristics. In the presented example *P_cr_* = 1/0.3949 = 2.5323 kN.

The critical forces, obtained experimentally, are presented in Table 2. Discrepancies between values shown in column 7 and values obtained by other methods are caused by the approximate character of the Southwell method for columns of variable cross-sections and the presence of material defects caused by the hot rolling and laser cutting of tested columns.

**Figure 14:**
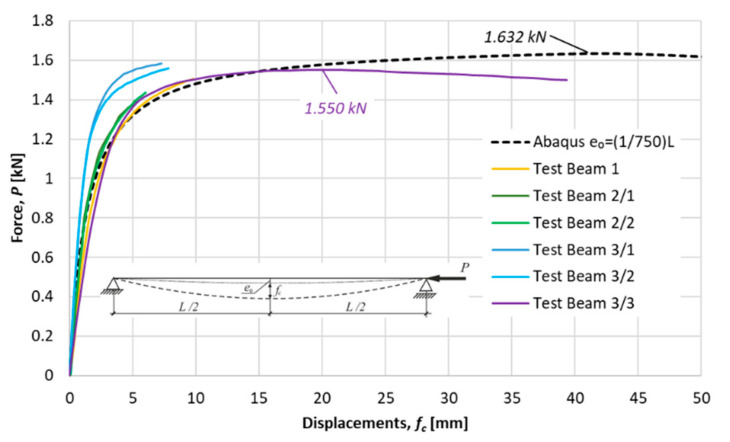
Specimen *L =* 1057.1 mm. Load deflection paths obtained in experimental tests and numerically.

**Figure 15:**
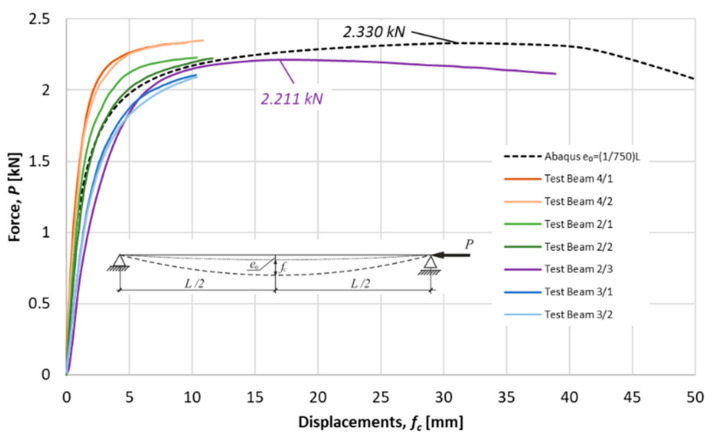
Specimen *L =* 915.1 mm. Load deflection paths obtained in experimental tests and numerically.

**Figure 16:**
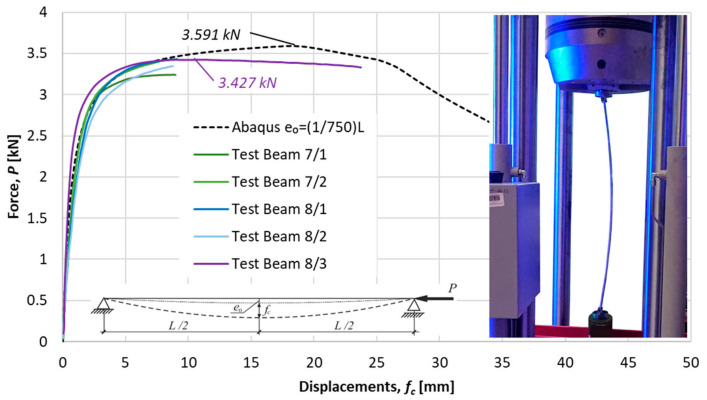
Specimen *L =* 765.1 mm. Load deflection paths obtained numerically and in experimental tests with the collapse mode.

Ultimate forces for tested specimens were detected as a maximum on load deflection characteristics obtained in experiments. The nonlinear load-displacements characteristics for the considered specimens are presented in Figure 14, Figure 15 and Figure 16. Besides the equilibrium paths obtained in experimental tests, also the equilibrium paths obtained numerically for the amplitude of bow imperfection $e_{0}=L/750$, are shown, as well.

Average measures of compression resistances obtained in experiments were equal 1.58 kN, 2.22 kN and 3.37 kN for columns of *L* = 1057.1, *L* = 915.1 and *L* = 765.1, respectively. These values are greater than resistances predicted by the procedure proposed in this paper (comp. column 4 in Table 7).

**Figure 17:**
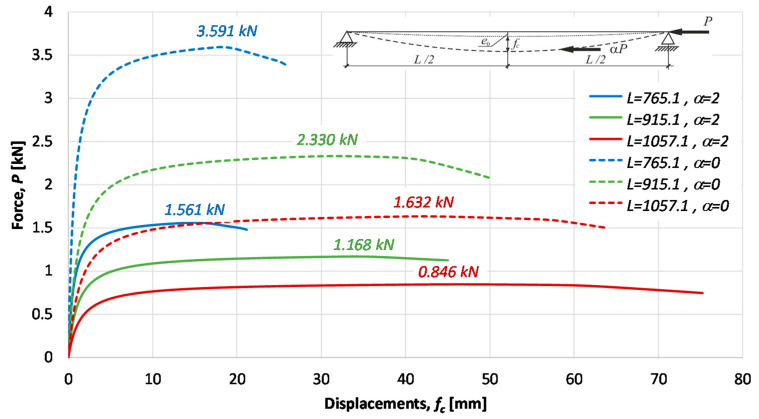
Load deflection paths obtained numerically for the initial bow imperfection of amplitude $e_{0}=L/750$.

Figure 17 presents collection of nonlinear equilibrium paths obtained numerically by means of Abaqus systems for the two families of considered columns, assuming the initial bow imperfection of amplitude $e_{0}=L/750$ and bilinear material model with the yield stress *f_y_* = 285 MPa and the strain hardening measured by the tangent modulus *E_tan_* = *E*/10^4^ (Figure 12). Maxima on obtained nonlinear equilibrium paths are measures of compression resistances. Obtained values are presented in column 5 of Table 7 (case *α* = 2) and in the column 5 of Table 8 (case *α* = 2).

Columns no. 6 in these tables express relations between compression resistances predicted by numerical analyses and design resistances proposed in the paper. In all the cases nearly the 10% margin was obtained.

## 5. Concluding Remarks

The presented paper provides closed formulae on critical forces acting on the two-segment stepped columns of general geometrical data and the stiffness distribution. The correctness of derived formulae was verified based on the exact analytical solution presented by Volmir [2] in the form of the transcendental equation. The additional comparisons to results of other authors and with numerical simulations were successful. It confirms usefulness of the derived formulae for the critical forces for engineering practice.

The derived formulae for the critical forces, complicated at the first glance, can be copied into a spreadsheet or into a software serving to symbolic computations. After substitution of specific values for particular parameters, corresponding to the given case of the two step compressed column, one can obtain the critical value of the force. Tables and nomograms presented in the paper can be additional assistance for quick assessment of critical forces for particular cases of stepped columns.

To estimate the buckling resistance of considered steel, stepped columns, the procedure based on classical Ayrton-Perry approach was presented. Particular steps of the procedure were similar to their counterparts adopted in provisions of Eurocode EN1993-1-1. Due to the fact that the cross-section is variable, the final formula for the buckling resistance must be applied at each section, and the smallest value is the searched measure of the column’s buckling resistance.

Experimental tests and numerical simulations have confirmed the correctness of the proposed procedures. Formulae on critical forces and the procedure leading to assessment of the buckling resistance, presented in the paper, can be a valuable assistance for designers engaged in the designing the two step columns.

## Figures and Tables

**Table 1:** Values *F_g_/F_d_* for different values *β* and *γ*.

	γ	0.00	0.10	0.20	0.30	0.40	0.50	0.60	0.70	0.80	0.90	1.00
β												
1.00		9.87	9.87	9.87	9.87	9.87	9.87	9.87	9.87	9.87	9.87	9.87
1.25		9.87	9.88	9.97	10.16	10.49	10.94	11.43	11.88	12.19	12.32	12.34
1.50		9.87	9.89	10.03	10.36	10.93	11.73	12.72	13.71	14.44	14.76	14.81
1.75		9.87	9.90	10.07	10.50	11.24	12.35	13.80	15.38	16.62	17.19	17.27
2.00		9.87	9.90	10.10	10.60	11.48	12.83	14.70	16.89	18.74	19.61	19.74
2.25		9.87	9.91	10.13	10.68	11.66	13.22	15.45	18.25	20.78	22.02	22.21
2.50		9.87	9.91	10.15	10.74	11.81	13.53	16.09	19.48	22.76	24.43	24.68
2.75		9.87	9.91	10.17	10.79	11.94	13.79	16.64	20.59	24.67	26.82	27.15
3.00		9.87	9.91	10.18	10.84	12.04	14.01	17.11	21.60	26.50	29.21	29.61
3.25		9.87	9.91	10.19	10.87	12.12	14.20	17.52	22.51	28.27	31.59	32.08
3.50		9.87	9.92	10.20	10.90	12.20	14.36	17.88	23.33	29.97	33.95	34.55
3.75		9.87	9.92	10.21	10.93	12.26	14.50	18.19	24.08	31.60	36.31	37.02
4.00		9.87	9.92	10.22	10.95	12.32	14.63	18.47	24.76	33.17	38.65	39.48

**Table 2:** Critical forces in [kN].

Column’s Case. Length in (mm)	*γ* = 607.6/L	Value Resulting from Equation (3)	$P_{cr}^{precise}$ Equation (6)	$P_{cr}^{num}$ COSMOS/M	$P_{cr}^{num}$ Abaqus	$P_{cr}^{exp}$ (Average)
L = 1057.1	0.575	1.687	1.686	1.686	1.684	1.532
L = 915.1	0.664	2.415	2.414	2.414	2.410	2.253
L = 765.1	0.794	3.721	3.720	3.722	3.718	3.490

**Table 3:** Values *P_g_/P_d_* for *α* = 0.5 and for different values *β* and *γ*.

	β	0.0	0.1	0.2	0.3	0.4	0.5	0.6	0.7	0.8	0.9	1.0
γ												
1.00		9.87	8.97	8.35	8.02	7.90	7.88	7.86	7.72	7.43	7.02	6.58
1.25		9.87	8.99	8.48	8.33	8.48	8.82	9.20	9.38	9.21	8.77	8.23
1.50		9.87	9.01	8.56	8.54	8.89	9.55	10.34	10.92	10.97	10.52	9.87
1.75		9.87	9.02	8.61	8.69	9.20	10.11	11.30	12.35	12.69	12.26	11.52
2.00		9.87	9.02	8.66	8.80	9.43	10.57	12.12	13.68	14.38	14.00	13.16
2.25		9.87	9.03	8.69	8.89	9.62	10.93	12.82	14.89	16.03	15.74	14.81
2.50		9.87	9.03	8.71	8.96	9.77	11.23	13.43	16.01	17.64	17.47	16.45
2.75		9.87	9.04	8.74	9.01	9.89	11.48	13.94	17.03	19.22	19.20	18.10
3.00		9.87	9.04	8.75	9.06	10.00	11.70	14.39	17.97	20.75	20.93	19.74
3.25		9.87	9.04	8.77	9.10	10.08	11.88	14.79	18.83	22.25	22.65	21.39
3.50		9.87	9.05	8.78	9.14	10.16	12.04	15.13	19.62	23.71	24.37	23.03
3.75		9.87	9.05	8.79	9.17	10.22	12.17	15.44	20.34	25.12	26.09	24.68
4.00		9.87	9.05	8.80	9.19	10.28	12.29	15.71	21.01	26.50	27.80	26.32

**Table 4:** Values *P_g_/P_d_* for *α* = 1.0 and for different values of *β* and *γ*.

	β	0.00	0.10	0.20	0.30	0.40	0.50	0.60	0.70	0.80	0.90	1.00
γ												
1.00		9.87	8.18	7.20	6.73	6.56	6.55	6.51	6.31	5.93	5.44	4.94
1.25		9.87	8.21	7.33	7.03	7.10	7.38	7.68	7.71	7.38	6.80	6.17
1.50		9.87	8.23	7.42	7.23	7.48	8.03	8.68	9.04	8.80	8.15	7.40
1.75		9.87	8.25	7.48	7.38	7.77	8.55	9.55	10.28	10.21	9.51	8.64
2.00		9.87	8.26	7.53	7.49	7.99	8.96	10.29	11.44	11.60	10.86	9.87
2.25		9.87	8.26	7.57	7.58	8.16	9.30	10.93	12.52	12.97	12.21	11.10
2.50		9.87	8.27	7.60	7.65	8.31	9.58	11.49	13.52	14.31	13.56	12.34
2.75		9.87	8.27	7.62	7.71	8.43	9.82	11.97	14.45	15.64	14.91	13.57
3.00		9.87	8.28	7.64	7.76	8.53	10.02	12.39	15.32	16.94	16.26	14.81
3.25		9.87	8.28	7.66	7.80	8.61	10.19	12.76	16.11	18.21	17.61	16.04
3.50		9.87	8.29	7.67	7.84	8.68	10.34	13.09	16.85	19.46	18.95	17.27
3.75		9.87	8.29	7.69	7.87	8.75	10.47	13.38	17.53	20.69	20.29	18.51
4.00		9.87	8.29	7.70	7.89	8.80	10.59	13.64	18.16	21.89	21.64	19.74

**Table 5:** Values *P_g_/P_d_* for *α* = 2.0 and for different values *β* and *γ*.

	β	0.0	0.1	0.2	0.3	0.4	0.5	0.6	0.7	0.8	0.9	1.0
γ												
1.00		9.87	6.91	5.59	5.06	4.89	4.88	4.83	4.61	4.21	3.75	3.29
1.25		9.87	6.94	5.73	5.32	5.33	5.55	5.75	5.67	5.25	4.68	4.11
1.50		9.87	6.97	5.82	5.51	5.65	6.08	6.55	6.68	6.28	5.62	4.94
1.75		9.87	6.99	5.89	5.64	5.90	6.51	7.26	7.65	7.31	6.55	5.76
2.00		9.87	7.00	5.94	5.75	6.09	6.86	7.87	8.57	8.32	7.49	6.58
2.25		9.87	7.01	5.97	5.83	6.25	7.15	8.41	9.44	9.33	8.42	7.40
2.50		9.87	7.02	6.01	5.90	6.37	7.39	8.89	10.26	10.33	9.36	8.23
2.75		9.87	7.03	6.03	5.95	6.48	7.59	9.30	11.03	11.32	10.29	9.05
3.00		9.87	7.03	6.05	6.00	6.57	7.77	9.67	11.75	12.30	11.22	9.87
3.25		9.87	7.04	6.07	6.04	6.64	7.92	9.99	12.43	13.26	12.16	10.69
3.50		9.87	7.04	6.09	6.07	6.71	8.05	10.28	13.06	14.22	13.09	11.52
3.75		9.87	7.05	6.10	6.10	6.77	8.16	10.53	13.66	15.16	14.02	12.34
4.00		9.87	7.05	6.11	6.12	6.82	8.27	10.76	14.21	16.09	14.95	13.16

**Table 6:** Critical forces in [kN] for the case *α* =2.

Column’s Case. Length in [mm]	γ = 607.6/L	Value Resulting from Equation (8)	$P_{cr}^{precise}$ Equation (18)	$P_{cr}^{num}$ COSMOS/M	$P_{cr}^{num}$ Abaqus
L = 1057.1	0.575	0.8727	0.8706	0.8708	0.8699
L = 915.1	0.664	1.2093	1.2026	1.2031	1.2020
L = 765.1	0.794	1.6319	1.6215	1.6226	1.6217

**Table 7:** Compressive resistance in [kN].

Column’s Case. Length in (mm)	γ = 607.6/L	Critical Force *P_cr_*	$P_{Rd}^{prop}$	$P_{Rd}^{num}$ Abaqus	col.5/col.4
L = 1057.1	0.575	1.687	1.483	1.632	1.10
L = 915.1	0.664	2.415	2.114	2.330	1.10
L = 765.1	0.794	3.721	3.257	3.591	1.10

**Table 8:** Compressive resistance in [kN] for the case *α* = 2.

Column’s Case. Length in (mm)	γ = 607.6/L	Critical Force *P_cr_*	$P_{Rd}^{prop}$	$P_{Rd}^{num}$ Abaqus	col.5/col.4
L = 1057.1	0.575	0.8727	0.7668	0.8460	1.10
L = 915.1	0.664	1.2093	1.060	1.1681	1.10
L = 765.1	0.794	1.6319	1.418	1.5613	1.10
